# The Effectiveness of Conjugate *Haemophilus influenzae* Type B Vaccine in The Gambia 14 Years After Introduction

**DOI:** 10.1093/cid/cit598

**Published:** 2013-09-17

**Authors:** Stephen R. C. Howie, Claire Oluwalana, Ousman Secka, Susana Scott, Readon C. Ideh, Bernard E. Ebruke, Anne Balloch, Sana Sambou, James Erskine, Yamundow Lowe, Tumani Corrah, Richard A. Adegbola

**Affiliations:** 1 Child Survival Theme; 2 Laboratory Services; 3 Disease Control and Elimination Theme, Medical Research Council Unit, Fajara, The Gambia; 4 Department of Infectious Disease Epidemiology, London School of Hygiene and Tropical Medicine, United Kingdom; 5 Infection and Immunity Theme, Murdoch Children's Research Institute, Parkville, Victoria, Australia; 6 Ministry of Health and Social Welfare, Banjul; 7 WEC Mission Hospital, Sibanor, The Gambia; 8 Directorate; 9 Bacterial Diseases Programme, Medical Research Council Unit, Fajara, The Gambia; 10 GlaxoSmithKline Vaccines, Wavre, Belgium

**Keywords:** Hib vaccination, Hib disease, surveillance, Gambia, Africa

## Abstract

***Background.*** The Gambia was the first country in Africa to introduce conjugate *Haemophilus influenzae* type b (Hib) vaccine, which, as in other developing countries but unlike industrialized countries, is delivered as a 3-dose primary series with no booster. This study assessed its effectiveness 14 years after introduction.

***Methods.*** Using methods standardized during >20 years in the study site, clinical and microbiological surveillance for invasive Hib disease (primarily meningitis) in the Western Region of The Gambia from 2007 to 2010 was complemented with studies of Hib carriage in children aged 1 to <2 years, Hib antibody levels in children aged <5 years, and Hib vaccine coverage and timing in children aged 1 to <2 years.

***Results.*** The incidence of Hib meningitis remained low (averaging 1.3 per 100 000 children aged <5 years annually), as did the Hib oropharyngeal carriage rate (0.9%). Hib antibody levels were protective in >99% of those surveyed, albeit with lower titers in older children; and coverage of conjugate Hib vaccination was high (91% having 3 doses at 1–2 years of age) using a schedule that was delivered at median ages of 2.6 months, 4.3 months, and 6 months for the first, second, and third doses, respectively.

***Conclusions.*** Conjugate Hib vaccine was delivered on time in a 3-dose primary series without booster to a high proportion of eligible children and this was associated with effective disease control up to 14 years after introduction. It is important that surveillance continues in this first African country to introduce the vaccine to determine if effective control persists or if a booster dose becomes necessary as has been the case in industrialized countries.

## Abstract (precis)

Fourteen years after the first introduction of conjugate *Haemophilus influenzae* type b vaccine in The Gambia, effective disease control was maintained, with associated low carriage and high seroprotection. Continued surveillance must determine if protection wanes and a booster dose is needed.

Invasive *Haemophilus influenzae* type b (Hib) disease, an important cause of meningitis, pneumonia and septicemia, is a major contributor to childhood morbidity and mortality in unvaccinated populations, most of which are in developing countries [1]. The Gambia was the first country in Africa to routinely vaccinate its children with conjugate Hib vaccine, beginning in 1997 using a primary series of 3 doses scheduled for children aged 2, 3, and 4 months and no booster dose, and this schedule remains unchanged. Using consistent surveillance methods focusing primarily on the detection of Hib meningitis, we reported the virtual disappearance of invasive Hib disease from The Gambia by 2002 [2], and its presence at a very low incidence thereafter [3]. The most pressing question for African and many other developing countries is whether a booster dose will be needed in the long term, and the experience in The Gambia is highly relevant to this question.

The rollout of Hib vaccine worldwide has been rapid in the last 15 years [4]. In 1997, just 15% of countries had introduced the vaccine [5], and >80% of countries worldwide were using it by 2010 [5], increasing to 92% in 2012 [6]. Importantly, >80% of GAVI Alliance support-eligible developing countries were using Hib vaccine by 2010 [7, 8]. Most industrialized countries have a booster dose in their routine schedule, but developing countries typically do not [9], which is consistent with World Health Organization (WHO) policy [10].

Apart from invasive Hib disease incidence data, vaccine coverage data, Hib carriage data, and community Hib antibody data give useful insights into the dynamics of Hib transmission and protection. In The Gambia, the prevalence of Hib carriage in children aged 1 to <2 years before routine vaccination was introduced was 12%, and this dropped to 0.25% by 2002. In 2000 the proportions of children aged 1 to <2 years having received 3 doses of vaccine were 68%, 2 doses 84%, and 1 dose 94%. The median age of children at vaccination was 3.4 months, 6.5 months, and 8 months for the first, second, and third doses, respectively. The vaccine preparation in which conjugate Hib vaccine is delivered changed midway through the surveillance period in June 2009 from quadrivalent diphtheria/tetanus/pertussis/Hib to pentavalent diphtheria/tetanus/pertussis/hepatitis B/Hib. Both preparations are from the Serum Institute of India and contain the PRP-T conjugated Hib antigen and whole cell pertussis. In addition to formal clinical and microbiological monitoring of disease in The Gambia between 2007 and 2010 [3], a number of other investigations of carriage, community seroprotection, and vaccine coverage and timing were done to explore the question of the vaccine's long-term effectiveness in this setting further, and these are reported here.

## MATERIALS AND METHODS

**Figure 1.**
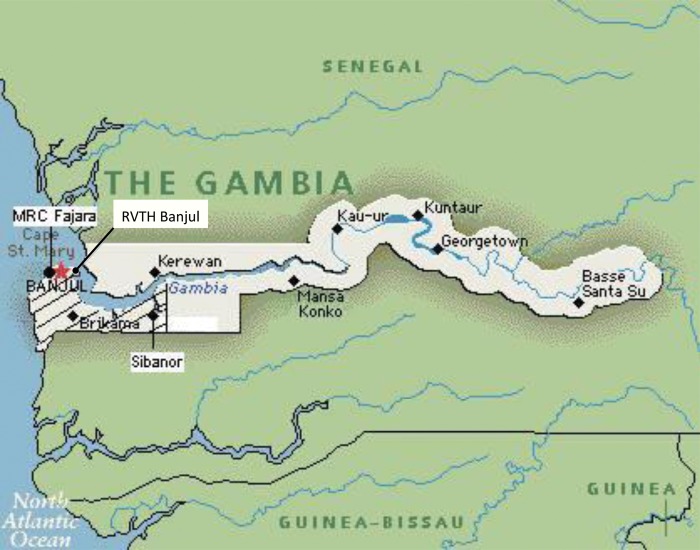
Map of The Gambia showing the Western Region study area (shaded) containing study hospitals in Fajara, Banjul, and Sibanor. Abbreviations: MRC, Medical Research Council; RVTH, Royal Victoria Teaching Hospital.

As elsewhere described [3], surveillance was carried out between 22 October 2007 and 31 December 2010 in the same area and using the same methods as used and reported previously [2]. Patients with suspected invasive Hib disease presenting to study hospitals were investigated by culture, and particular emphasis was placed on the detection of Hib meningitis by culture of cerebrospinal fluid and blood. Surveillance was undertaken in the Western Region of The Gambia (Figure 1), which had a total population of 836 000 in the 2003 census (60% of the population of The Gambia) and comprises urban, periurban, and rural areas. The population aged <5 years in this area was 100 000 in 2003 (census data) and was estimated to have a mean of 128 000 in the calendar years 2008–2010.

In addition to disease surveillance, investigations of Hib carriage, community Hib antibody levels, and Hib vaccine coverage were undertaken, and the methods for these are described here.

### Carriage Study

One thousand children aged 1 to <2 years, 500 each from urban (Fajikunda and Serekunda) and rural (Sibanor) well-child clinics, were investigated for Hib carriage, using the same methods as previously used [2],. All children in the target age range presenting to the clinic were eligible for recruitment regardless of vaccination status. These children had oropharyngeal swabbing done by 2 trained field workers. The swabs were plated onto Hib antiserum plates and placed in standard fashion in a candle-containing jar and transported to the microbiology laboratory at the Medical Research Council Unit, Fajara. We estimated that 874 participants would be required to detect a rise of carriage prevalence from 0.25% (the 2000–2001 level) to 2% with a power of 90% and a 5% significance level. New carriage rates were compared with carriage rates obtained in 2000–2001 [2].

### Antibody Survey

Hib antibody levels were measured for 3 different age groups (1 to <2 years, 2 to <3 years, and 3 to <5 years) to assess immunity by age. The aim was to recruit 250 children in each group, giving 90% power at a 5% significance level to detect a difference in proportion of children having a protective antibody level (>0.15 µg/mL) of 70% in the 1- to <2-year age group and 55% in either of the other groups.

Hib antibody assays were performed at the Murdoch Children's Research Institute, Victoria, Australia, following established methods [11–14]. Microtiter plates were coated with *H. influenzae* Type B Oligosaccharide–Human Serum Albumin Conjugate (BEI Resources, Manassas, Virginia). The standard, anti-Hib capsular polysaccharide serum (lot 1983; FDA, Kensington, Maryland), control anti-Hib human reference serum (National Institute for Biological Standards and Control, UK), and patient samples were incubated on precoated plates. Horseradish peroxidase–conjugated anti-human immunoglobulin G (Millipore, Australia) and a tetramethylbenzidine (TMB) substrate solution (KPL, Gaithersburg, Maryland) were subsequently added to the plates for detection. The optical density of each well was read on the microplate reader at 450 nm (reference filter 630 nm). Optical density data were converted to antibody concentrations with KCjunior software (Bio-Tek Instruments Inc). Results were calculated using a standardized curve-fitting 4-parameter logistic method.

Logarithms of Hib antibody levels and geometric mean antibody titers and their 95% confidence intervals (CIs) were calculated. Logistic and linear regression was used to estimate differences in Hib seropositivity (as defined by >0.15 or 1 µg/mL) and log titers between areas of residence and age groups. Statistical analyses were performed using Stata statistical software version 12.0 (StataCorp, College Station, Texas).

### Vaccine Coverage and Timing

Vaccine coverage was assessed in 1 to <2 years olds using the cluster survey technique recommended by WHO [15]. A cluster was defined as ≥7 children in the target age range. The coverage estimate has a 95% confidence level of ±10%. The survey, which consisted of visiting homes and examining immunization records of children, was carried out in June 2010 in 120 clusters, 30 clusters being systematically selected in each of the Banjul, Kanifing, Kombos, and Fonis districts across the study area. Fieldworkers visited at least 7 children's homes in each cluster to collect data on their immunization status against measles, yellow fever, polio, tuberculosis, tetanus, diphtheria, pertussis, hepatitis B, and Hib.

## RESULTS

**Figure 2.**
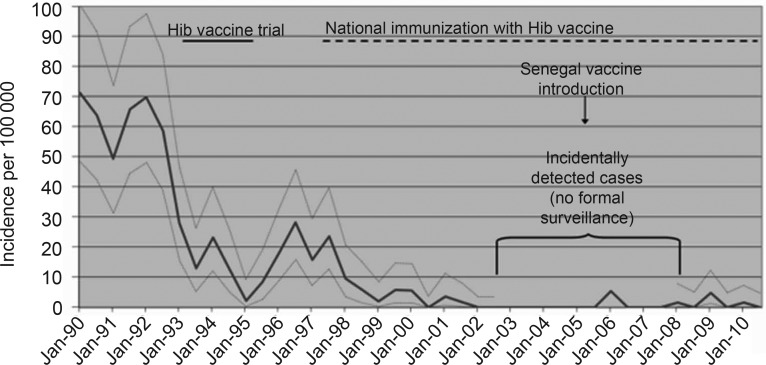
Incidence of *Haemophilus influenzae* type b (Hib) meningitis in children <5 years of age, cases per 100 000 per year, in the Western Region of The Gambia from 1990 to 2010.

Details of the incidence of invasive Hib disease in the study period, which remained low at an average of 1.3 cases of Hib meningitis per 100 000 children aged <5 years annually, have been reported elsewhere (Figure 2) [3]. The total number of cases seen was 9, with 3 having had ≥2 doses of vaccine, 4 having 0–1 dose, and 2 having an unknown number of doses. Four cases occurred before the introduction of the pentavalent preparation midway through the study period and 5 after. The median age of patients was 5 months (range, 3–13 months), and they were evenly divided between meningitis cases (n = 5) and septicemia cases with no evidence of meningitis (n = 4).

### Hib Carriage

**Figure 3.**
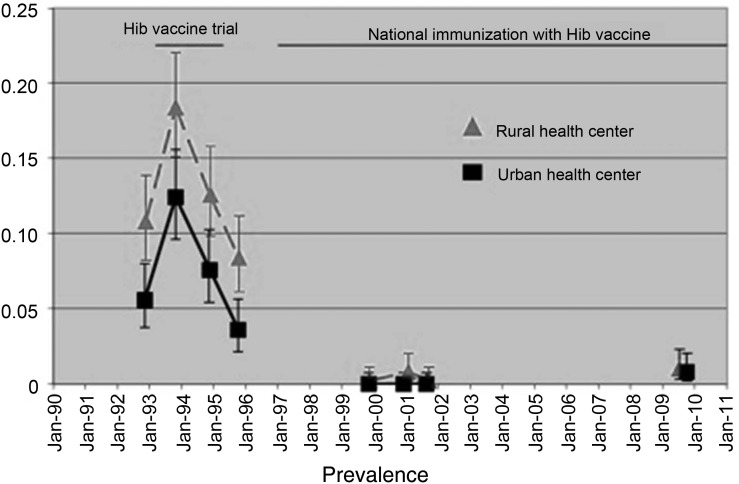
Temporal trends in *Haemophilus influenzae* type b (Hib) carriage in children aged 12–23 months in rural (Sibanor) and urban (Serekunda/Fajikunda) settings.

The carriage study was conducted between April 2009 and February 2010. The median age of the children recruited was 18.5 months (range, 12–22 months). Nine isolates of Hib were obtained from oropharyngeal swabs, giving a carriage prevalence of 0.9% (95% CI, .3%–1.5%), compared to 0.25% (95% CI, .06%–.9%) in 2002 (*P* = .28). Figure 3 shows current and historical carriage rates in urban and rural locations.

### Hib Antibodies

The Hib antibody survey was carried out between May 2009 and April 2010. Seven hundred sixty-two children were included, 419 (55%) rural (Sibanor) and 343 (45%) urban (Serekunda and Fajikunda). Across the 3 age bands, the numbers enrolled were 239 (31%) aged 1 to <2 years, 249 (33%) aged 2 to <3 years, and 272 (36%) aged 3 to <5 years. Two children had missing dates of birth, so their ages could not be calculated. Overall, 99.3% of the participants had antibody concentrations of ≥0.15 µg/mL, and proportions were similar across age groups and areas of residence. The overall geometric mean Hib antibody concentration was 2.25 (95% CI, 2.07–2.45). The details of antibody levels by age and area of residence are shown in Table 1. There were no differences between the 2 urban sites, so their data have been combined. Higher levels of antibodies to Hib were associated with urban compared to rural residency; Hib antibody levels in the urban areas were 42% (95% CI, 20%–68%; *P* < .001) higher than those at the rural area. Antibody levels to Hib were 22% (95% CI, 4%–36%; *P* = .02) lower in the 3- to <5-year age group compared to those in the 1- to <2-year age group. There was no evidence for a difference in antibody levels to Hib between those aged 1 to <2 years and those aged 2 to <3 years (*P* = .09). Overall, 75% (95% CI, 72%–78%) had concentrations >1 µg/mL. The proportion with Hib antibody concentrations >1 µg/mL were also higher for those from the urban areas compared to the rural area (adjusted odds ratio [AOR], 1.60 [95% CI, 1.14–2.26], *P* = .007). No difference was observed between the proportions fully vaccinated in urban (98.5%) and rural (97.5%) groups (*P* = .32). Being older was associated with lower odds of concentrations >1 µg/mL (AOR for 2 years vs 1 year, 0.6 [95% CI, .39–.94], and 3 years vs 1 year, 0.5 [95% CI, .31–.72]).

**Table 1.** Hib Antibody Levels in 762 Gambian Children by Age and Area of Residence

Characteristic	No.	% ≥0.15 µg/mL	(95% CI)	% >1 µg/mL	(95% CI)	Adjusted^a^ OR for >1 µg/mL	(95% CI)	*P* Value	GMT	(95% CI)	Adjusted^a^ % Difference in GMT	(95% CI)	*P* Value
Overall	762	99	(98–100)	75	(72–78)				2.248	(2.07–2.45)	…	…	
Area of residence													
Rural^b^	419	99	(97–100)	71	(66–75)	Baseline	…		1.913	(1.71–2.14)	Baseline	…	
Urban^b^	343	100	(99–100)	80	(76–84)	1.60	(1.14–2.26)	.007	2.740	(2.42–3.11)	41.93	(19.86–68.06)	<.001
Age group, y^c^													
1 to <2	239	99	(97–100)	83	(77–87)				2.628	(2.27–3.05)	Baseline	…	
2 to <3	249	100	(98–100)	75	(69–80)	0.60	(.39–.94)	.025	2.208	(1.91–2.55)	−16.63	(−32.36 to 2.77)	.088
3 to <5	272	99	(97–100)	69	(63–74)	0.47	(.31–.72)	<.001	2.002	(1.73–2.32)	−21.89	(−36.38 to −4.11)	.018

Abbreviations: CI, confidence interval; GMT, geometric mean titer; hib, *Haemophilus influenzae* type b; OR, odds ratio.

^a^ Adjusted by other variables in the table.

^b^ Rural: Sibanor; urban: Serekunda and Fajikunda combined.

^c^ Two children with missing ages.

### Hib Vaccine Coverage and Timing

Nine hundred thirty-two children aged 1 to <2 years were visited in 120 clusters in the Western Health Region of The Gambia in June 2010. All but 1 of the 932 children surveyed had their vaccination record cards available. Overall, 91% of the children had received 3 doses of Hib vaccine, 96% 2 doses, and 98% 1 dose. The median age of children at the first, second, and third doses was 2.6 months (interquartile range [IQR], 2.2–3.3 months), 4.3 months (IQR, 3.6–5.1 months), and 6.0 months (IQR, 5.0–7.5 months,) respectively.

## DISCUSSION

In the period 2007–2010, the incidence of Hib meningitis and all invasive Hib disease remained low in the context of a routine primary series of conjugate Hib vaccine without a booster. Vaccine coverage was >90% for DTP3 (3 doses of diphtheria-tetanus-pertussis vaccines) and the doses were given without substantial delay. Hib carriage remained low but not absent at 0.9% in children aged 1 to <2 years. Hib antibody levels were at or above a putative protective level of 0.15 µg/mL in >99% of children aged <5 years surveyed, although a decline in antibody levels with advancing age was noted. These data point to ongoing effective control and the need for vigilance to ensure this continues.

Although almost all industrialized countries have introduced Hib vaccine with a booster in addition to a primary series [16, 17], there are other experiences of good control from a primary series alone, including in African countries. In South Africa, the incidence of disease reduced markedly after vaccine introduction in 1999 [18]. Between 2003 and 2009, invasive Hib disease increased, albeit at modest levels, from 0.7 to 1.3 per 100 000 children aged <5 years [19]. A booster dose was introduced in 2010 in combination with inactivated polio vaccine, but polio control was the driver for this, not Hib disease. The incidence of Hib meningitis reported from Kenya in children aged <5 years decreased, from 71 to 8 per 100 000 after vaccine introduction in 2001 [20], and has not risen since (Anthony Scott, written personal communication, March 2013). The vaccine was introduced into Malawi and Uganda in 2002, and although no population-based incidence estimates are available, the reported number of hospital cases markedly decreased, and had been sustained through 2009–2010 [9, 21]. Chile, Colombia, and Brazil have all maintained good control without a booster dose, with vaccine introductions occurring in 1996, 1998, and 1999, respectively [22–24].

Good long-term control, however, has not been the experience of all countries that have started with a 3-dose primary series alone. The United Kingdom introduced Hib vaccine in 1993 and was one of the few industrialized countries not to schedule a booster dose from the outset. Control was excellent initially [25] and carriage was eliminated [26], but after several years a resurgence of disease prompted the introduction of a booster dose in 2003, which was associated with a return to good control [25]. The introduction of a new and less immunogenic formulation including acellular pertussis vaccine may have played a role in waning immunity [27]. The United Kingdom differed from The Gambia in a number of potentially relevant respects also, including by having a lower preintroduction Hib carriage prevalence and a higher median age of disease and by accompanying introduction with a catch-up campaign [28]. Ireland appears to have had a similar experience to the United Kingdom [29]. A continuing postintroduction low level of Hib carriage and transmission in The Gambia, not seen in the United Kingdom, may provide an immunological stimulus that boosts community protection and reduces the chances of a booster dose being required.

The putative protective level of Hib antibody is regarded as being 0.15 µg/mL [30], and no differences were observed between sites or age groups for the proportions of participants with these levels, which were extremely high. Some regard a level of >1 µg/mL as being indicative of longer-term protection, although this is not clear-cut [13, 14]. At this higher level, which was achieved in 75% of children overall, some differences were observed, most notably that this proportion dropped from 83% in 1-year-olds to 69% in 3- and 4-year-olds. The median age of cases in The Gambia is <12 months [2, 3], but were the epidemiology of invasive Hib disease to change by an increase in the age of cases, this waning might become important. The reason for the observed difference in antibody levels between urban and rural areas is not clear. Differences in environmental factors and host factors, such as nutritional status, may play a part, although this study did not address this question. A higher prevalence of HIV infection in the rural area of the study has been observed [31] but the significance of this for community Hib seroprotection is unknown. What is also unknown is whether the observed difference has public health significance, a question that can be addressed with continuing surveillance.

A potential weakness of this study is that it is likely that cases have been missed, a weakness shared by all surveillance studies. Hospital-based surveillance underrepresents the incidence of disease in the community, as not all patients will seek hospital care, and this is particularly so for less severe disease. The efficacy of Hib vaccine has been shown to be similar against Hib meningitis and Hib pneumonia; while Hib meningitis is the form of invasive Hib disease most reliably diagnosed using routine clinical management supported by sound bacteriology [32]. In view of this, an emphasis on meningitis cases has been a deliberate measure to minimize fluctuations in the sensitivity of surveillance and give as robust an indication of long-term patterns of control as possible. Although antibody concentrations were assessed, antibody avidity was not, and this may have provided a further insight into community seroprotection. A key strength of this study is that it used the same surveillance methods as previously used in this study area [2], and this has yielded comparable data spanning 20 years (Figure 1). Additionally, all the methods used for the carriage, serology, and vaccine coverage studies were accepted standard methods that have been used in previous studies here, enhancing the comparability of data through time.

The high proportion of countries (93% in 2012) [6] that have introduced conjugate Hib vaccination does not reflect the proportion of the world's children being protected by Hib vaccine, which is much lower. This is primarily because several high-burden, high-population countries have yet to introduce the vaccine, notably China and the Russian Federation, or have not yet introduced the vaccine universally, such as in Nigeria and India [4]. In Nigeria and India the vaccine has recently been introduced into some regions after years of debate and advocacy [4, 33–36]. It is currently estimated that 45% of the world's infants live in countries that have not introduced the vaccine, and 52% of infants worldwide are not receiving Hib vaccine [4]. Protecting these children remains a top global health priority.

The experience of invasive Hib disease control in The Gambia 14 years after vaccine introduction is encouraging. The good control seen in African countries introducing the vaccine after The Gambia is also encouraging. The global experience of conjugate Hib vaccine shows that it has been a highly successful public health intervention [37]. However, it is well recognized that the effectiveness of control can wane, and for this reason it is very important that surveillance be maintained for its global as well as local relevance.

## CONCLUSIONS

In the period of this study, no evidence emerged to support the introduction of a booster dose of conjugate Hib vaccine into the Gambian Expanded Programme on Immunisation (EPI). The incidence of invasive Hib disease remained low (average 1.3 Hib meningitis episodes per 100 000 children aged <5 years annually); the Hib nasopharyngeal carriage rate in children aged 1 to <2 years remained low (0.9%); Hib antibody levels in young children were high, with >99% of those surveyed having protective levels; and coverage of conjugate Hib vaccination was high (91% having 3 doses at 1 to <2 years of age) using a primary 3-dose schedule that was confirmed to be a primary series in practice as well as in theory. A decline in antibody levels with age was observed. These data together confirm that the EPI was delivering the primary conjugate Hib vaccine series on time to a high proportion of eligible children and that this was associated with effective disease control in the period studied. It is important that surveillance be continued to determine if effective control persists or whether control and immunity wane and a booster dose becomes necessary.
